# Prevalence of Congenital Anomalies in the Japan Environment and Children’s Study

**DOI:** 10.2188/jea.JE20180014

**Published:** 2019-07-05

**Authors:** Hidetoshi Mezawa, Ai Tomotaki, Kiwako Yamamoto-Hanada, Kazue Ishitsuka, Tadayuki Ayabe, Mizuho Konishi, Mayako Saito, Limin Yang, Narufumi Suganuma, Fumiki Hirahara, Shoji F. Nakayama, Hirohisa Saito, Yukihiro Ohya, the Japan Environment and Children’s Study Group

**Affiliations:** 1 Medical Support Center for the Japan Environment and Children’s Study, National Center for Child Health and Development, Tokyo, Japan; 2 Department of Environmental Medicine, Kochi Medical School, Kochi, Japan; 3 Department of Obstetrics and Gynecology, Yokohama City University School of Medicine, Kanagawa, Japan; 4 Japan Environment and Children’s Study Programme Office, National Institute for Environmental Studies, Tsukuba, Ibaraki, Japan

**Keywords:** congenital anomalies, Japan Environment and Children’s Study, JECS, prevalence, birth cohort

## Abstract

**Background:**

The aims of the present report were to estimate the prevalence of congenital anomalies (CAs) among infants in Japan using data from the Japan Environment and Children’s Study (JECS) and to evaluate the validity of CA classification within JECS.

**Methods:**

Data on CAs were collected at delivery and at age 1 month from the medical records of 101,825 infants at 15 regional centers. The analyses focused on 61 CAs, selected on the basis of reported associations with environmental exposure. Prevalence per 10,000 pregnancies (including miscarriages, stillbirths, and live births) was stratified according to four reporting patterns (at delivery, at age 1 month, at either, and at both). To evaluate the accuracy of observed CA prevalence, the medical records of 179 cases from a single JECS regional center underwent independent, retrospective re-evaluation.

**Results:**

The prevalence of major CAs in four reporting patterns (at delivery, at age 1 month, at either, and at both) was 2.4, 2.6, 3.5, and 1.4 for myelomeningocele/spina bifida; 4.3, 4.2, 5.3, and 3.2 for cleft palate; 18.1, 17.4, 19.5, and 15.1 for cleft lip with or without cleft palate; 73.4, 100.3, 120.8, and 52.8 for congenital heart disease; and 10.5, 14.1, 15.0, and 9.6 for Down’s syndrome, respectively. In the subsample re-evaluation, CA diagnoses were confirmed for 92.7%, 93.3%, 90.5%, and 97.8% of cases in the four reporting patterns (at delivery, at age 1 month, at either, and at both), respectively.

**Conclusions:**

The present report generated reliable data concerning the prevalence of major CAs in JECS.

## INTRODUCTION

Congenital anomalies (CAs) are an important cause of not only infant and childhood deaths but also chronic illness and lifelong disability.^1^ To date, approximately 50% of CAs have been linked to a specific cause, genetic, socioeconomic and demographic, environmental, infectious, and maternal nutritional risk factors are known at present.^2^ Environmental toxicants that are known to cause several CAs are one of the risk factors that can be minimized by appropriate standards and regulations. The reported risk factors for congenital heart disease include prenatal exposure to pesticides, organic solvents, and air pollution.^3^^,^^4^ Exposure to some endocrine disrupters has been linked to cryptorchidism and hypospadias.^5^ Pesticide exposure is associated with urinary malformations.^6^ However, evidence for many of these relationships is still limited.

The Japan Environment and Children’s Study (JECS) is a nationwide prospective birth cohort study conducted by the Ministry of the Environment of Japan. The aims of JECS are to identify environmental factors, in particular chemical substances, with a detrimental impact on child health and development, and to facilitate improved environmental risk management system.

CAs are one of the priority health outcomes of JECS. However, the collection of CA data in JECS is hampered by the fact that, to date, no population-based CA registration or surveillance system has been established in Japan. At its 15 regional centers, JECS investigators therefore collect data on CAs from the medical records of infants at delivery and at 1 month of age.

The aims of the present report were to estimate the prevalence of CAs among infants from Japan using data from the JECS cohort and to evaluate the validity of CA classification within JECS.

## METHODS

### Study design

Recruitment of women in the first trimester of pregnancy took place between January 2011 and March 2014 at the 15 JECS regional centers. The respective offspring will undergo follow-up until the age of 13 years. JECS protocols are described in detail elsewhere.^7^^,^^8^ During the JECS recruitment period, a total of 104,102 infants were enrolled through hospitals or local government offices. In the event of miscarriage, medical termination of pregnancy, stillbirth, or neonatal mortality, the respective regional center collected and recorded the necessary data prior to termination of JECS participation. All types of obstetric facilities (perinatal medical centers, maternity hospitals, and maternity homes) cooperated with JECS to increase the participation rate. No data concerning the participation rate across the entire JECS recruitment period were collected, since the recruitment area was expanded in 2012 and recruitment during 2011 and 2014 was restricted to a period of 3 months. However, in 2013, when recruitment had largely stabilized, JECS collected data on 45% of all total live births across its 15 geographical recruitment regions.^7^

All JECS procedures adhere to the Ethical Guidelines for Epidemiological Research of the Japan Ministry of Health, Labour, and Welfare. JECS protocols were reviewed and approved by the Institutional Review Board of the Ministry of the Environment, and by the ethics committees of all participating institutions. Written informed consent was obtained from all pregnant women prior to inclusion.

### Cohort used for the investigation of CA prevalence

For the purpose of the present report, the following JECS cases were excluded: (i) cases in which no data at the CA reporting time-points were available (*n* = 2,277), and ii) cases in which pregnancy outcome was unknown (*n* = 38).

Some participants were withdrawn from the study after the completion of data collection at delivery due to miscarriage (*n* = 1,340) and stillbirth (*n* = 291). Follow-up of these participants was terminated following the collection of data at delivery. In 705 participants, data were available at the delivery time-point but not at 1 month. In the case of 44 participants, data were available at the 1 month time-point but not at delivery. In each case, the reason for this was undocumented.

### Collection of congenital anomaly data

The present analyses focused on a list of 61 CAs (Table 1). This represents a modified version of the list used in the Hokkaido Study on Environment and Children’s Health. The latter comprised 55 CAs, which were selected as possible markers of environmental exposure.^9^

**Table 1.** Number and percentage of the 61 congenital anomalies of interest according to reporting and data collection patterns

	ICD-10 code^c^	Data collected at delivery only								Data collected at one month of age only		Data collected both at delivery and at one month of age							

		Spontaneous abortion		Medical termination of pregnancies		Stillbirth		Live birth		Live birth		Reported at delivery only		Reported at one month only		Reported either at delivery or at one month		Reported both at delivery and at one month	
*N*		1,010		330		291		705		44		99445							
Anencephaly	Q00	3	0.297	12	3.636	0	0.000	1	0.142	0	0.000	12	0.012	5	0.005	16	0.016	1	0.001
Encephalocele	Q01	1	0.099	1	0.303	0	0.000	1	0.142	0	0.000	10	0.010	12	0.012	19	0.019	3	0.003
Microcephaly	Q02	0	0.000	1	0.303	0	0.000	0	0.000	0	0.000	10	0.010	28	0.028	37	0.037	1	0.001
Hydrocephaly	Q03	1	0.099	2	0.606	2	0.687	3	0.426	1	2.273	34	0.034	63	0.063	80	0.080	17	0.017
Craniotabes	P96.3	0	0.000	0	0.000	0	0.000	0	0.000	0	0.000	35	0.035	35	0.035	69	0.069	1	0.001
Holoprosencephaly	Q04.2	2	0.198	7	2.121	1	0.344	1	0.142	0	0.000	12	0.012	27	0.027	32	0.032	7	0.007
Agenesis of corpus callosum	Q04.0	0	0.000	2	0.606	1	0.344	0	0.000	1	2.273	13	0.013	15	0.015	20	0.020	8	0.008
Ablepharon	Q10.3	0	0.000	0	0.000	0	0.000	0	0.000	0	0.000	5	0.005	8	0.008	13	0.013	0	0.000
Anophthalmos/microphthalmos	Q11.0–Q11.2	0	0.000	0	0.000	0	0.000	1	0.142	0	0.000	15	0.015	11	0.011	21	0.021	5	0.005
Congenital cataract	Q12.0	0	0.000	0	0.000	0	0.000	0	0.000	0	0.000	17	0.017	13	0.013	30	0.030	0	0.000
Microtia	Q17.2	0	0.000	0	0.000	0	0.000	0	0.000	0	0.000	19	0.019	24	0.024	40	0.040	3	0.003
Congenital aural atresia	Q16.1	1	0.099	0	0.000	0	0.000	0	0.000	0	0.000	32	0.032	16	0.016	39	0.039	9	0.009
Cryptotia	Q17.8	0	0.000	0	0.000	0	0.000	0	0.000	0	0.000	19	0.019	19	0.019	33	0.033	5	0.005
Low set ears	Q17.4	3	0.297	2	0.606	3	1.031	8	1.135	0	0.000	54	0.054	36	0.036	78	0.078	12	0.012
Cleft lip	Q36	1	0.099	3	0.909	1	0.344	1	0.142	0	0.000	63	0.063	62	0.062	76	0.076	49	0.049
Cleft palate	Q35	1	0.099	0	0.000	0	0.000	1	0.142	0	0.000	42	0.042	41	0.041	52	0.052	31	0.031
Cleft lip and palate	Q37.0–Q37.5	1	0.099	0	0.000	2	0.687	5	0.709	0	0.000	107	0.108	101	0.102	117	0.118	91	0.092
not classified as cleft lip, cleft palate or cleft lip and palate^a^																13	0.013		
Facial cleft	Q37.8 Q37.9	0	0.000	0	0.000	0	0.000	0	0.000	0	0.000	7	0.007	4	0.004	9	0.009	2	0.002
Natal teeth	K00.6	0	0.000	0	0.000	0	0.000	0	0.000	0	0.000	46	0.046	32	0.032	64	0.064	14	0.014
Diaphragmatic hernia	Q79.0	0	0.000	0	0.000	1	0.344	5	0.709	0	0.000	28	0.028	22	0.022	37	0.037	13	0.013
Intralobar sequestration	Q33.2	0	0.000	0	0.000	0	0.000	0	0.000	0	0.000	1	0.001	4	0.004	5	0.005	0	0.000
Congenital cystic adenomatoid malformation	Q33.0	0	0.000	0	0.000	0	0.000	1	0.142	0	0.000	9	0.009	11	0.011	14	0.014	6	0.006
Pulmonary hypoplasia	Q33.6	0	0.000	0	0.000	1	0.344	6	0.851	0	0.000	17	0.017	19	0.019	27	0.027	9	0.009
Congenital heart disease	Q20–Q28	3	0.297	3	0.909	4	1.375	33	4.681	0	0.000	704	0.708	978	0.983	1187	1.194	495	0.498
Cardiac arrhythmia	I44, I45, I47, I48, I49	0	0.000	0	0.000	0	0.000	2	0.284	0	0.000	80	0.080	48	0.048	104	0.105	24	0.024
Omphalocele	Q79.2	2	0.198	4	1.212	0	0.000	1	0.142	0	0.000	36	0.036	265	0.266	282	0.284	19	0.019
Gastroschisis	Q79.3	4	0.396	4	1.212	1	0.344	2	0.284	0	0.000	9	0.009	5	0.005	10	0.010	4	0.004
Esophageal atresia with or without fistula	Q39.0 Q39.1	0	0.000	0	0.000	1	0.344	4	0.567	0	0.000	14	0.014	14	0.014	18	0.018	10	0.010
Duodenal atresia/stenosis	Q41.0	0	0.000	0	0.000	0	0.000	0	0.000	0	0.000	17	0.017	13	0.013	19	0.019	11	0.011
Intestinal atresia/stenosis	Q41.1–Q41.9	0	0.000	0	0.000	0	0.000	0	0.000	0	0.000	16	0.016	10	0.010	18	0.018	8	0.008
Anorectal atresia/stenosis	Q42.0–Q42.9	0	0.000	0	0.000	0	0.000	1	0.142	0	0.000	38	0.038	36	0.036	45	0.045	29	0.029
Inguinal hernia	K40.2	0	0.000	0	0.000	0	0.000	0	0.000	0	0.000	29	0.029	98	0.099	113	0.114	14	0.014
Congenital hydronephrosis	Q62.0	0	0.000	1	0.303	0	0.000	0	0.000	0	0.000	145	0.146	163	0.164	216	0.217	92	0.093
Cystic kidney	Q61	0	0.000	2	0.606	0	0.000	4	0.567	0	0.000	23	0.023	22	0.022	31	0.031	14	0.014
Renal agenesis	Q60.2	2	0.198	1	0.303	2	0.687	0	0.000	0	0.000	5	0.005	6	0.006	8	0.008	3	0.003
Hypospadias	Q54	0	0.000	0	0.000	0	0.000	1	0.142	0	0.000	46	0.046	50	0.050	65	0.065	31	0.031
Undescended testis/cryptorchidism	Q53	0	0.000	0	0.000	0	0.000	2	0.284	0	0.000	213	0.214	161	0.162	306	0.308	68	0.068
Bladder exstrophy	Q64.1	0	0.000	0	0.000	0	0.000	0	0.000	0	0.000	3	0.003	2	0.002	3	0.003	2	0.002
Enlarged clitoris	N90.8	0	0.000	0	0.000	0	0.000	0	0.000	0	0.000	6	0.006	6	0.006	12	0.012	0	0.000
Abnormal Vaginal Opening	Q52.4	1	0.099	0	0.000	0	0.000	0	0.000	0	0.000	1	0.001	1	0.001	2	0.002	0	0.000
Indeterminate sex	Q56	1	0.099	0	0.000	0	0.000	1	0.142	0	0.000	6	0.006	2	0.002	7	0.007	1	0.001
Polydactyly of fingers	Q69 Q70.4	0	0.000	3	0.909	2	0.687	0	0.000	0	0.000	98	0.099	82	0.082	106	0.107	74	0.074
Syndactyly of fingers	Q70	0	0.000	2	0.606	0	0.000	4	0.567	0	0.000	34	0.034	25	0.025	41	0.041	18	0.018
Cleft hand	Q71.6	0	0.000	0	0.000	0	0.000	0	0.000	0	0.000	4	0.004	4	0.004	5	0.005	3	0.003
Polydactyly of toes	Q69 Q70.4	0	0.000	0	0.000	0	0.000	1	0.142	0	0.000	67	0.067	57	0.057	88	0.088	36	0.036
Syndactyly of toes	Q70	0	0.000	0	0.000	0	0.000	3	0.426	0	0.000	70	0.070	73	0.073	101	0.102	42	0.042
Cleft foot	Q72.7	0	0.000	0	0.000	0	0.000	0	0.000	0	0.000	6	0.006	4	0.004	7	0.007	3	0.003
Hemangioma	Q82.5	0	0.000	0	0.000	0	0.000	1	0.142	0	0.000	186	0.187	633	0.637	756	0.760	63	0.063
Epidermolysis bullosa	Q82.3	0	0.000	0	0.000	0	0.000	1	0.142	0	0.000	9	0.009	7	0.007	14	0.014	2	0.002
Myelomeningocele/Spina bifida	Q05	0	0.000	1	0.303	1	0.344	1	0.142	0	0.000	21	0.021	23	0.023	33	0.033	11	0.011
Down syndrome	Q90	0	0.000	9	2.727	0	0.000	6	0.851	0	0.000	92	0.093	129	0.130	138	0.139	83	0.083
Trisomy 18	Q91.0–Q91.3	0	0.000	5	1.515	7	2.405	6	0.851	0	0.000	19	0.019	23	0.023	27	0.027	15	0.015
Trisomy 13	Q91.4–Q91.7	2	0.198	0	0.000	1	0.344	0	0.000	0	0.000	3	0.003	5	0.005	5	0.005	3	0.003
Turner syndrome	Q96	1	0.099	1	0.303	0	0.000	0	0.000	0	0.000	2	0.002	3	0.003	4	0.004	1	0.001
Thanatophoric dysplasia	Q77.1	0	0.000	2	0.606	0	0.000	1	0.142	0	0.000	2	0.002	1	0.001	3	0.003	0	0.000
Acrodysostosis, not specificied	Q77.4	0	0.000	0	0.000	1	0.344	0	0.000	0	0.000	4	0.004	3	0.003	5	0.005	2	0.002
Developmental dysplasia of the hip^b^	Q65.0–Q65.6									0	0.000			14	0.014	14	0.014		
Congenital multiple arthrogryposis	Q74.3	0	0.000	0	0.000	0	0.000	0	0.000	0	0.000	5	0.005	4	0.004	8	0.008	1	0.001
Floppy infant	P94.2	0	0.000	0	0.000	0	0.000	0	0.000	0	0.000	11	0.011	8	0.008	16	0.016	3	0.003
Conjoined twins	Q89.4	1	0.099	1	0.303	0	0.000	0	0.000	0	0.000	0	0.000	2	0.002	2	0.002	0	0.000
Amniotic band constriction	Q79.8	3	0.297	4	1.212	0	0.000	0	0.000	0	0.000	2	0.002	2	0.002	2	0.002	2	0.002

ICD-10, International Statistical Classification of Diseases and Related Health Problems, 10th revision.

Each congenital anomaly is listed with its number of cases and percentage per data collection patterns.

^a^“not classified as cleft lip, cleft palate or cleft lip and palate” included the different reporting case from at delivery and at one month of age within cleft lip, cleft palate or cleft lip and palate.

^b^Developmental dysplasia of the hip was included in the transcription form at 1 month of age only.

^c^Transcriptions of both the ICD-10 code and congenital anomaly name in medical records were collected at the data collection phase.

CA data were collected by transcribing information entered in the infants’ medical records at delivery and at 1 month of age onto JECS transcription forms. This information included the respective 10^th^ revision of the International Statistical Classification of Diseases and Related Health Problems (ICD) code (Table 1), and the terminology used to denote the CA in the medical records. In each case, this process was completed by a physician, a midwife, a nurse, or a trained research coordinator. Each CA was listed in the transcription form in a separate box. If any of the 61 CAs of interest were observed, a tick was entered into the corresponding box.

### Categorization of anomalies described in the comment section

When CAs not listed in Table 1 were found or suspected in medical records, the information was transcribed into a comment section (ie, uncategorized CAs), to reduce the number of cases overlooked by the person making the transcription.

In total, 13,144 uncategorized CAs were recorded in the comment sections. Two pediatricians (H.M. and Y.Y.) independently evaluated the respective comments and categorized the described anomalies as specific CAs. If uncategorized CA information containing misspellings, unexplained abbreviations, or any ambiguity were detected, these were corrected by the pediatricians prior to categorization. For the purposes of the present analyses, suspected CAs were assessed using the comment sections of the transcription forms only. The respective medical records were not consulted. Suspected CAs still awaiting formal diagnosis were not categorized as CAs for the purposes of the present analyses. In cases of uncertainty, the two pediatricians assigned a consensus diagnosis.

For the valuation of entries in the comments section, 31 CAs that were easily detected at delivery and that generally required prompt medical attention after delivery were selected from the list of 61 CAs of interest. The 31 selected CAs comprised anencephaly, encephalocele, hydrocephaly, holoprosencephaly, ablepharon, microphthalmia/anophthalmia, congenital cataract, cleft lip (CL), cleft palate (CP), cleft lip and palate (CLP), facial cleft, diaphragmatic hernia, congenital heart disease, omphalocele, gastroschisis, esophageal atresia with or without fistula, duodenal atresia/stenosis, intestinal atresia/stenosis, anorectal atresia/stenosis, hypospadias, undescended testis/cryptorchidism, polydactyly of the fingers, syndactyly of the fingers, cleft hand, polydactyly of the toes, syndactyly of the toes, cleft foot, myelomeningocele/spina bifida, Down syndrome, Trisomy 18, and Trisomy 13. Through this process, a total of 755 uncategorized CAs (5.74%) were categorized as CAs.

### Covariates

Data were also collected on mother’s age at delivery, regional center, singleton/multiple pregnancy, and pregnancy outcomes (live birth, spontaneous abortion, medical termination of pregnancy, and stillbirth). Stillbirth was defined as the occurrence of fetal death at >21 weeks gestation.

### Analysis of CA prevalence

Data collected at delivery only were stratified according to four pregnancy outcomes: 1) spontaneous abortion, 2) medical termination of pregnancy, 3) stillbirth, and 4) live birth. All cases in which CAs had been recorded at the 1 month time-point only were classified as live births.

In a first step, data collected both at delivery and at 1 month were stratified according to the four possible CA reporting patterns: 1) CA recorded at delivery time-point only, 2) CA recorded at 1 month time-point only, 3) CA recorded either at delivery or at 1 month, and 4) CA recorded at both delivery and the 1 month time-point. For each of these four categories, the type, number, and percentage of CAs are shown in Table 1. A high CA prevalence was observed in the following two data collection patterns: 1) data collected at delivery only, and 2) data collected at 1 month of age only. The number of CAs recorded in these two data collection patterns are shown in Table 2.

**Table 2.** Number and prevalence per 10,000 pregnancies of the 61 congenital anomalies of interest according to reporting pattern

Congenital anomaly	Total number				Prevalence per 10,000 pregnancies			
	
	D-DELIVERY^a^	D-1M^b^	D-OR^c^	D-AND^d^	D-DELIVERY^a^	D-1M^b^	D-OR^c^	D-AND^d^
Total number	101,825	101,825	101,825	101,825				
Major anomalies	1,959	2,437	3,041	1,290	192.4	239.3	298.6	126.7

Central nervous system	174	228	313	84	17.1	22.4	30.7	8.2
Neural tube defect	65	61	88	37	6.4	6.0	8.6	3.6
Anencephaly	28	21	32	17	2.7	2.1	3.1	1.7
Encephalocele	13	15	22	6	1.3	1.5	2.2	0.6
Myelomeningocele/Spina bifida	24	26	36	14	2.4	2.6	3.5	1.4
Hydrocephaly	43	72	89	26	4.2	7.1	8.7	2.6
Microcephaly	11	29	38	2	1.1	2.8	3.7	0.2
Holoprosencephaly	23	38	43	18	2.3	3.7	4.2	1.8
Craniotabes	35	35	69	1	3.4	3.4	6.8	0.1
Agenesis of corpus callosum	17	19	24	12	1.7	1.9	2.4	1.2

Eye	33	24	50	6	3.2	2.4	4.9	0.6
Anophthalmos/microphthalmos	16	12	22	6	1.6	1.2	2.2	0.6
Congenital cataract	17	13	30	0	1.7	1.3	2.9	0.0

Ear	51	35	71	15	5.0	3.4	7.0	1.5
Congenital aural atresia	33	17	40	10	3.2	1.7	3.9	1.0
Cryptotia	19	19	33	5	1.9	1.9	3.2	0.5

Oro-facial cleft	232	223	254	188	22.8	21.9	24.9	18.5
Cleft palate	44	43	54	33	4.3	4.2	5.3	3.2
Cleft lip with or without palate^e^	184	177	199	154	18.1	17.4	19.5	15.1
Facial cleft	7	4	9	2	0.7	0.4	0.9	0.2

Respiratory system	35	40	52	23	3.4	3.9	5.1	2.3
Intralobar sequestration	1	4	5	0	0.1	0.4	0.5	0.0
Congenital cystic adenomatoid malformation	10	12	15	7	1.0	1.2	1.5	0.7
Pulmonary hypoplasia	24	26	34	16	2.4	2.6	3.3	1.6

Congenital heart disease	747	1,021	1,230	538	73.4	100.3	120.8	52.8
Cardiac arrhythmia	82	50	106	26	8.1	4.9	10.4	2.6

Abdominal wall defects	61	286	307	40	6.0	28.1	30.1	3.9
Omphalocele	43	272	289	26	4.2	26.7	28.4	2.6
Gastroschisis	20	16	21	15	2.0	1.6	2.1	1.5

Digestive system	121	100	140	79	11.9	9.8	13.7	7.8
Esophageal atresia with or without fistula	19	19	23	15	1.9	1.9	2.3	1.5
Duodenal atresia/stenosis	17	13	19	11	1.7	1.3	1.9	1.1
Intestinal atresia/stenosis	16	10	18	8	1.6	1.0	1.8	0.8
Anorectal atresia/stenosis	39	37	46	30	3.8	3.6	4.5	2.9
Diaphragmatic hernia	34	28	43	19	3.3	2.7	4.2	1.9

Urinary system	186	201	265	122	18.3	19.7	26.0	12.0
Congenital hydronephrosis	146	164	217	93	14.3	16.1	21.3	9.1
Cystic kidney	29	28	37	20	2.8	2.7	3.6	2.0
Renal agenesis	10	11	13	8	1.0	1.1	1.3	0.8
Bladder exstrophy	3	2	3	2	0.3	0.2	0.3	0.2

Genital system	53	55	73	35	5.2	5.4	7.2	3.4
Hypospadias	47	51	66	32	4.6	5.0	6.5	3.1
Indeterminate sex	8	4	9	3	0.8	0.4	0.9	0.3

Limb	232	212	273	158	22.8	20.8	26.8	15.5
Polydactyly	162	137	184	111	15.9	13.5	18.1	10.9
Polydactyly of fingers	103	87	111	79	10.1	8.5	10.9	7.8
Polydactyly of toes	68	58	89	37	6.7	5.7	8.7	3.6
Syndactyly	104	99	135	64	10.2	9.7	13.3	6.3
Syndactyly of fingers	40	31	47	24	3.9	3.0	4.6	2.4
Syndactyly of toes	73	76	104	45	7.2	7.5	10.2	4.4
Cleft hand or foot	9	6	10	5	0.9	0.6	1.0	0.5
Cleft hand	4	4	5	3	0.4	0.4	0.5	0.3
Cleft foot	6	4	7	3	0.6	0.4	0.7	0.3

Skeletal dysplasia	10	8	12	6	1.0	0.8	1.2	0.6
Thanatophoric dysplasia	5	4	6	3	0.5	0.4	0.6	0.3
Acrodysostosis, not specificied	5	4	6	3	0.5	0.4	0.6	0.3

Chromosomal	154	195	208	140	15.1	19.2	20.4	13.7
Down syndrome	107	144	153	98	10.5	14.1	15.0	9.6
Trisomy 18	37	41	45	33	3.6	4.0	4.4	3.2
Trisomy 13	6	8	8	6	0.6	0.8	0.8	0.6
Turner syndrome	4	5	6	3	0.4	0.5	0.6	0.3

Ablepharon	5	8	13	0	0.5	0.8	1.3	0.0
Epidermolysis bullosa	10	8	15	3	1.0	0.8	1.5	0.3
Developmental dysplasia of the hip^f^	—	14	14	—	—	1.4	1.4	—
Congenital multiple arthrogryposis	5	4	8	1	0.5	0.4	0.8	0.1
Floppy infant	11	8	16	3	1.1	0.8	1.6	0.3
Conjoined twins	2	4	4	2	0.2	0.4	0.4	0.2
Amniotic band constriction	9	9	9	9	0.9	0.9	0.9	0.9

Minor anomalies								
Microtia	19	24	40	3	1.9	2.4	3.9	0.3
Low set ears	70	52	94	28	6.9	5.1	9.2	2.7
Natal teeth	46	32	64	14	4.5	3.1	6.3	1.4
Inguinal hernia	29	98	113	14	2.8	9.6	11.1	1.4
Undescending testis/cryptorchidism	215	163	308	70	21.1	16.0	30.2	6.9
Enlarged clitoris	6	6	12	0	0.6	0.6	1.2	0.0
Abnormal Vaginal Opening	2	2	3	1	0.2	0.2	0.3	0.1
Hemangioma	187	634	757	64	18.4	62.3	74.3	6.3

^a^D-DELIVERY includes congenital anomalies reported only at the birth data collection phase.

^b^D-1M includes congenital anomalies reported only at one month data collection.

^c^D-OR includes congenital anomalies reported either at birth or at one month data collection.

^d^D-AND includes congenital anomalies reported both at birth and at one month data collection, only at birth, and only at one month data collection.

^e^CL and CLP were re-categorized as ‘cleft lip with or without cleft palate’ (CL/P).

^f^Developmental dysplasia of the hip was only collected at one month data collection.

In a second step, data from the two data collection patterns showing a high prevalence of CAs (delivery only and 1 month of age only) were stratified for the four CA reporting patterns: 1) CA reporting at delivery data collection only (D-DELIVERY), 2) CA reporting at 1 month data collection only (D-1M), 3) CA reporting either at delivery or at 1 month data collection (D-OR), and 4) CA reporting both at delivery and at 1 month data collection (D-AND). For each of the four reporting patterns, the prevalence of the CA per 10,000 pregnancies was then calculated.

### Categorization of CP, CL, and CLP

In accordance with other epidemiological studies, CL and CLP were re-categorized as ‘cleft lip with or without cleft palate’ (CL/P). In eight cases, CLP was reported at one time and CL at another time. In four cases, CL and CLP were reported at the each time. In one case, CP and CL were reported at the each time. Therefore, 13 cases were recorded as “not classified as CL, CP, or CLP” in Table 1. Of these, eight cases were categorized as CL/P for the purposes of the present analyses.

### Independent evaluation of congenital anomaly data accuracy

For almost all CAs, a difference in prevalence was observed between the four reporting categories. From December 2016 through March 2017, the accuracy of data collection was therefore assessed via direct, expert re-evaluation of transcription form content. For this purpose, three physicians (two pediatricians and one physician) worked with the Kochi regional center, and each hospital pediatrician and maternal clinic gynecologist independently performed a retrospective review of the transcription forms and respective medical and health insurance records of 179 CA infants from the Kochi regional center. In total, the Kochi regional center enrolled 7,140 pregnant women. The target anomalies for this re-evaluation process were the 31 easily detectable, major CAs assessed in the analysis of comment section data. The 179 infants were all cases with the target anomalies.

In cases in which information on the CA could not be located in the medical or insurance records, the physicians categorized the transcription form as erroneous and added a comment to the data. The re-evaluation results were categorized as: 1) confirmed or 2) not confirmed. The number and percentage of CAs in each category were then calculated according to the data reporting pattern.

### Statistical analysis

All analyses were performed using SAS version 9.4 (SAS Institute, Cary, NC, USA). Binary variables are shown as the number of persons and percentages (%) by category. Incidence is the total number of each CA case in the data reporting pattern. CA cases categorized according to organ were counted if any subordinate CAs were reported. In this previous report, prevalence estimated per 10,000 pregnancies (including spontaneous abortion, medical termination of pregnancy, stillbirth, and live birth) was stratified according to the reporting pattern. All prevalence included any type of data collection.

## RESULTS

**Figure 1.**
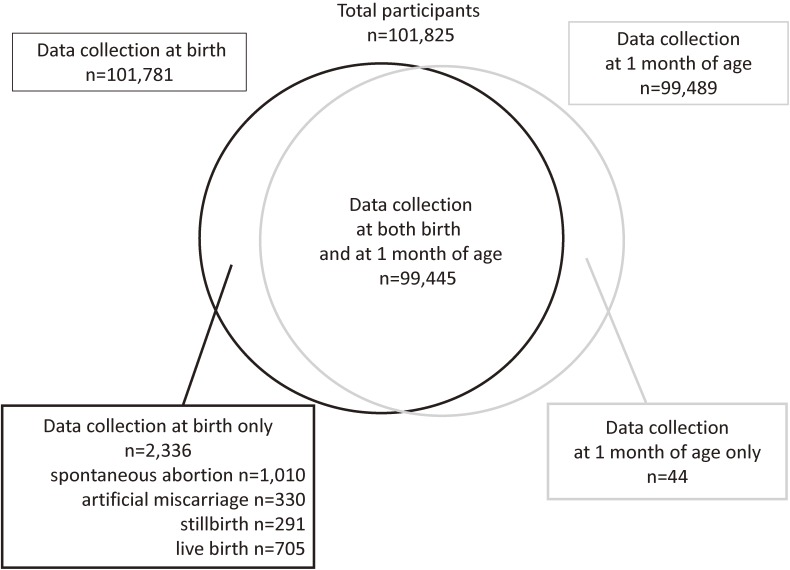
Venn diagram of congenital anomaly data collected from information entered in the medical records at delivery and at 1 month of age.

The final analyses included data from a total of 101,825 infants. Figure 1 shows the Venn diagram of the three data collection patterns: (i) both at delivery and at 1 month of age (*n* = 99,445 infants), (ii) at delivery only (*n* = 2,336 infants), and (iii) at 1 month of age only (*n* = 44 infants). Pregnancy outcomes in the “at delivery only group” comprised 291 stillbirths (0.29%), 1,010 spontaneous abortions (0.99%), 330 medical terminations of pregnancies (0.32%), and 705 livebirths (0.69%). The numbers of infants at each regional center, stratified according to maternal age at delivery, are shown in eTable 1.

Table 1 shows the number and percentages of 61 CAs of interest, stratified according to reporting pattern. In the spontaneous abortion, medical termination of pregnancy, and stillbirth groups, several major CAs were reported. In particular, nine Down syndrome cases (2.73%) were found in the medical termination of pregnancy group, while six Down syndrome cases (0.85%) were reported in the live birth group.

Among live births in the group with at delivery data only, major CAs were reported in 705 infants. Two major CAs were also reported in the group with 1 month data only. Among data collected both at delivery and at 1 month, the total number of each CA varied according to data reporting pattern, with the exception of amniotic band constriction. The number of omphalocele cases was substantially higher at 1 month than at delivery.

### Prevalence of each congenital anomaly

Table 2 summarizes the number and prevalence per 10,000 pregnancies of the 61 CAs of interest, stratified according to reporting pattern. The prevalence of major anomalies in each reporting pattern ranged from 126.7 to 298.6 per 10,000 pregnancies. D-OR had the highest and D-AND the lowest prevalence. Compared with D-DELIVERY and D-1M, the prevalence of almost all major CAs was similar or higher in D-1M. Concerning prevalence according to organ, congenital heart disease showed the highest prevalence, followed by ∼20 cases per 10,000 pregnancies for limb, urinary system, oro-facial cleft, and central nervous system. By contrast, the prevalence of eye, ear, respiratory system, genital system, and skeletal dysplasia CAs was low, at less than 10 per 10,000 pregnancies. The prevalence of digestive system and chromosomal CAs was 10 per 10,000 pregnancies. There was a difference in the number of abdominal wall defects between D-DELIVERY (6.0) and D-1M (28.1).

### Independent evaluation of congenital anomaly data accuracy

Of the 179 CA cases that underwent re-evaluation, one was found to have been miscoded in the transcription form, eight were withdrawn from the study after delivery, and two were not recorded due to the hospital’s refusal to disclose the information. Details of the remaining 168 cases are shown in Table 3. In the D-DELIVERY group, 115 CAs (92.7%) were confirmed and nine (7.3%) were not confirmed. In the D-1M group, 126 CAs (93.3%) were confirmed and nine (6.7%) were not confirmed. In the D-OR group, 152 CAs (90.5%) were confirmed and 16 (9.5%) were not confirmed. In the D-AND group, 89 CAs (97.8%) were confirmed and two (2.2%) were not confirmed. For 16 cases, no description of the CA was found in the respective medical or and health insurance records. The 16 suspected CAs comprised diaphragmatic hernia (*n* = 5), congenital heart disease (*n* = 3), congenital hydronephrosis (*n* = 2), duodenal atresia/stenosis (*n* = 1), intestinal atresia/stenosis (*n* = 1), myelomeningocele/spina bifida (*n* = 1), omphalocele (*n* = 1), polydactyly of the fingers (*n* = 1), and undescended testis/cryptorchidism (*n* = 1). Of the five cases of unreported diaphragmatic hernia, four were in the D-DELIVERY and one was in the D-1M. There were no other CAs without descriptions in the medical and insurance records in the non-confirmed 16 cases. eTable 2 shows the number and proportion of CAs for which confirmation in accordance with the ICD-10 code entered in the respective medical records was achieved.

**Table 3.** Number and percentage of congenital anomalies in the Kochi subsample according to reporting pattern

		D-DELIVERY^a^		D-1M^b^		D-OR^c^		D-AND^d^	
Medical records	Confirmed	115	92.7%	126	93.3%	152	90.5%	89	97.8%
	Not confirmed	9	7.3%	9	6.7%	16	9.5%	2	2.2%

Total		124	100%	135	100%	168	100%	91	100%

Each medical record status is shown with number and percentage by category.

^a^D-DELIVERY includes congenital anomalies reported only at delivery data collection.

^b^D-1M includes congenital anomalies reported only at 1 month data collection.

^c^D-OR includes congenital anomalies reported either at delivery or at 1 month data collection.

^d^D-AND includes congenital anomalies reported both at delivery and at 1 month data collection, only at delivery, and only at 1 month data collection.

## DISCUSSION

Using data from the JECS nationwide prospective birth cohort study, the present report analyzed: (i) data collection patterns, and (ii) the number and prevalence of 61 CAs of interest, which were stratified according to the pattern of data reporting. A high CA prevalence was observed in data collected at a single time-point (at delivery only or at 1 month of age only). In addition, differing numbers of CAs were observed between the different data reporting patterns. To investigate this, an independent evaluation was performed of the accuracy of the transcribing of data from medical records to the study transcription forms across each of the data reporting patterns. This was performed using a subsample from the Kochi regional JECS center. In this evaluation, confirmation in the medical/insurance records was achieved for 90.5% of CAs in the D-OR and 97.8% of CAs in the D-AND. We also reported differences in prevalence according to organ.

In the present report, at least 90.5% of the CAs reported in the transcription forms were confirmed after re-evaluation of the corresponding medical and insurance records. Transcription errors were mainly due to misclassifications that caused a bias toward the null value when the misclassification rate was equal between the two compared groups.^9^ In the JECS protocol, the misclassification was equal for exposures to environmental toxicants because the assessors were blinded to the exposure measurements. Rui et al investigated the impact of misclassification and measurement errors using a simulation study approach. In their study, when the prevalence of one disease was low (3.2%) and the effect size was small (0.3), a power of 15.1% was observed between no misclassification and high sensitivity and specificity scenario (sensitivity 0.84 and specificity 0.96) with a small amount of covariate measurement error.^10^ By contrast, the JECS sample size was calculated with sufficient power. For instance, to test a hypothesis concerning a disorder with a prevalence of 0.1%, such as Down syndrome, with a relative risk of 2.0 and an alpha error of 0.05, and using a cohort in which the proportion of individuals with a high level of exposure to the chemical substance of interest is 25%, a sample size of 64,536 participants is required to provide a statistical power of 80%.^11^ The JECS cohort exceeds this sample size by 101,825. Thus, even if around 15% of the statistical power was lost, the JECS CA data still retained sufficient power to address the study hypotheses. Thus, since data accuracy in the Kochi report had a sensitivity of >0.84 and a specificity of >0.96 during the transcription procedure, the JECS had sufficient accuracy in reporting CAs.

In the data accuracy report of the D-OR group, the Kochi regional center enrolled a total of 7,140 infants. Of these, 168 (2.4%) reported CAs and 6,972 (97.6%) did not. In the reported CA group, 152 (2.1%) were confirmed and 16 (0.2%) were not. Because of the high number of non-reported CA infants, our report could not provide an accuracy rate for non-reported infants. However, other mistakable CAs diagnosis in respective medical and insurance record were not found among non-confirmed cases. This might suggest a risk of mistakes in using the transcription forms. The number of transcription errors for non-reported CAs infants was expected to be at least similar to the number of non-confirmed CAs (16 cases). Using a sensitivity of >0.84 and a specificity of >0.96, the number of transcription errors in cases in the non-reported group was calculated to be 28 (sensitivity 0.84 and specificity 0.99). This number was more than expected. Taking this into consideration, the JECS CA data can be considered to have provided acceptable power for detecting the impact of environmental factors on the CA occurrence. By contrast, while D-OR had the highest number of CAs, it also had the highest rate of misclassification. This misclassification would be expected to cause an underestimation or an overestimation of CA prevalence. eTable 2 provides evidence of overestimation from the ICD-10 code. However, infants born in maternal homes were not diagnosed with any CAs using the ICD-10 code because midwives are not qualified to use the ICD-10 code in Japan. For these reasons, the present authors recommend the use of two different CA reporting groups in future analyses of CA prevalence, for example, D-1M and D-OR.

In the medical termination of pregnancy and stillbirth groups, a high proportion of life-threatening CAs and chromosomal diseases were found. Schechtman et al reported a direct correlation between CA severity and the rate of medical termination of pregnancy rates.^12^ Furthermore, a study in Japan showed that medical termination of pregnancy was performed in 93.3% of cases in which a chromosomal anomaly was confirmed via prenatal conventional cytogenetic analysis at 15–18 weeks of pregnancy.^13^ However, in the present report, life-threatening CAs were also recorded in the spontaneous abortion group. These comprised three cases of anencephaly, three cases of gastroschisis, and one case of an omphalocele. Severe CAs were also recorded in live births from the group with at delivery data only. These included CLP, hydrocephaly, diaphragmatic hernia, and esophageal atresia with or without fistula. Among the 44 cases for whom data were collected at 1 month of age only, two CAs were recorded. These results suggest that JECS participants with only a single data collection time-point (ie, at delivery only or at 1 month only) may have a high CA risk. To account for this, CA prevalence was determined using data collected both at delivery and at 1 month.

To describe the CA data characteristics, we compared the CAs in our previous study with those in other registrations. Previous estimates of CP and CL/P prevalence per 10,000 births were 5.83 and 22.76, respectively, in the 2012 report of the Japanese Association of Obstetricians and Gynecologists (JAOG)^14^; 5.88 and 8.26, respectively, in the 2011–2015 survey of the European Surveillance of Congenital Anomalies (EUROCATS)^15^; and 6.35 and 10.63, respectively, in the 2004–2006 survey of the National Birth Defects Prevention Network (NBDPN) in the United States.^16^ CP prevalence in each of these three reports was similar to, or slightly higher than, that found in the present report. Compared to the present report, CL/P prevalence in EUROCATS and NBDPN were lower, while CL/P prevalence in JAOG was higher. For spina bifida, the prevalence in the present analyses was lower than the prevalence reported in JAOG (5.18) and EUROCATS (5.11) and similar to or lower than the prevalence reported in NBDPN (3.50).

These cross-study similarities and differences in the prevalence of these major CAs are attributable to three factors. First, the findings are influenced by selection bias. The research facilities of the JAOG are mainly based at hospitals that provide care for women with high-risk pregnancies, whereas the JECS collects data from pregnant women who are managed in general hospitals and maternity homes. Since spina bifida requires surgery immediately after delivery, women with spina bifida pregnancies are usually referred to tertiary centers.^17^ By contrast, surgery for newborns with CP and CL/P is undertaken around 3–6 months after delivery.^18^ In the Hokkaido study, prenatal diagnosis rates for spina bifida were 100%, whereas they were approximately 81.8% and 92% for CP and CL/P.^19^ Moreover, between 2006 and 2016, 22 cases (31.0%) were referred after delivery to the Yamaguchi University Hospital.^20^ This meant that CP and CL/P newborns were mainly delivered at hospitals, as well as at maternity homes. These differences led to higher prevalence rates for spina bifida, CP, and CL/P in JAOG. Second, the cohorts investigated in EUROCATS, NBDPN, and the present report differed in terms of genetic factors. CL/P prevalence varies according to race/ethnicity. Prevalence is lowest in American blacks (5.8/10,000 live births), highest in American Indian or Alaska Natives (14.3/10,000 live births), and intermediate in other groups (whites, Hispanics, Asians, and Pacific Islanders).^21^ Moreover, non-syndromic CL/P has been associated with a number of genetic mutations,^22^^–^^24^ and the frequency of single nucleotide polymorphism risk variants for non-syndromic CL/P differs according to race/ethnicity.^25^ By contrast, there are no definite genetic risk factors for CP or spina bifida. EUROCATS did not analyze race/ethnicity rates. However, the cohort covered 29% of births in Europe. Thus, the cohort may have been multiethnic in nature. NBDPN involved American blacks, American Indian or Alaska Natives, whites, Hispanics, Asians, and Pacific Islanders. By contrast, the JECS cohort was almost exclusively comprised of individuals from the Japanese population. These genetic factors may partly explain the difference in CL/P prevalence between the three reports. To investigate these differences, genetic analyses of individuals in the JECS cohort are warranted. Third, the three cohorts showed differences in terms of environmental factors. Folic acid deficiency is a well-known risk factor for spina bifida,^26^ and a possible risk factor for CL/P but not for CP.^27^ Additional risk factors for CL/P include smoking,^27^^,^^28^ alcohol,^29^ and drugs.^30^^,^^31^ Known risk factors for spina bifida include pre-gestational diabetes,^32^ obesity,^33^ drugs,^34^ and pesticide exposure.^35^ However, research has generated inconsistent findings for CA associations with these risk factors, in particular environmental toxicants, and most of the studies were conducted in Europe and the United States. However, Europe, the United States, and Japan have obvious geographic, nutritional, and cultural differences, and the translation of findings from non-Asian countries to the Japanese population is problematic. Further studies from JECS would provide important data concerning the association between environmental exposures and CAs in Asian countries.

Omphalocele prevalence in JAOG, EUROCATS, and NBDPN was 2.22, 3.38, and 1.86, respectively, per 10,000 births. The present finding of 26.6 per 10,000 pregnancies in D-1M was therefore substantially higher than expected. This is attributable to the Japanese terminology for this particular CA. ‘Saitai-hernia,’ which is the Japanese term for ‘omphalocele’, is easily confused with ‘Sai-hernia,’ or ‘umbilical hernia.’ Umbilical hernia is a common condition in infants, with a prevalence of 1.9–18.5% in Caucasian populations.^36^ Thus, the occurrence in D-OR and D-1M is not appropriate for the analysis of omphalocele prevalence.

The prevalence of congenital heart disease was also higher in the present cohort than in EUROCAT. The prevalence in EUROCATS was 76.46. In JECS, data on all forms of congenital heart disease were collected under the broad category “congenital heart disease.” This included low severities of congenital heart disease, such as patent ductus arteriosus and patent foramen ovale. By contrast, these low severity heart diseases were excluded from EUROCATS. This might have resulted in the higher prevalence of congenital heart disease in JECS. For future investigations of specific congenital anomalies, such as tetralogy of Fallot, JECS plans to access other databases, such as the database of congenital heart disease in the information center for specific pediatric chronic diseases in Japan.

JECS collected CA data at delivery and at 1 month of age, but the protocol can result in unidentified CA cases being ignored. For example, the Western Australian Birth Defects Registry collected data from 3,294 CA cases born in 2000 and 2001. These subjects had been diagnosed up to 6 years of age: 616 (18.7%) were diagnosed prenatally, 1,574 (47.8%) by 1 month of age, 671 (20.4%) between 2 months and 11 months, and 400 (12.1%) between 1 and 6 years of age.^37^ For comparison, the British Isles Network of Congenital Anomaly Registers collected data for 5,911 CA cases born in 2012. These subjects had been diagnosed up to 2 years of age: 3,074 (52%) were diagnosed prenatally, 1,291 (21.8%) after delivery until 1 month of age, and 835 (14%) at an unknown time-point. After excluding cases for whom the timing of diagnosis was unknown, 4,365 (86%) of cases were diagnosed before 1 month of age.^38^ These reports showed an increased rate of prenatal CA diagnosis as a result of prenatal screening. In Japan, the guidance given to pregnant women at regular health checks recommends testing by ultrasound at least four times: twice before 23 weeks gestational age, once between 24 and 35 weeks, and once after 36 weeks. In the United Kingdom and Australia, prenatal ultrasound screening is performed twice, at around 12 and 20 weeks gestational age. The CA detection rate of JECS was at least as high as that of the United Kingdom in 2012. As expected, approximately 70–80% of CAs cases in JECS were diagnosed before the age of 1 month.

The present report has three main strengths. First, JECS CA data are based on reports from hospitals and maternity clinics throughout Japan. Reporting bias was not considered, since data collection was performed by research facility personnel rather than by parents. Second, the rate of loss to follow-up was low (2.2%). Third, CA prevalence was estimated on the basis of all pregnancies in the cohort, including all cases of spontaneous abortion and medical termination of pregnancy. Such data, in particular those concerning spontaneous abortion, are not recorded in other CA registries.^15^^,^^16^^,^^37^^,^^38^ However, these data are important in terms of the identification of the environmental causes of CAs.

The present report also had four limitations that warrant mention. First, only the 61 CAs of interest were considered. Second, misclassification may have occurred due to the mistranscription of medical records, which could have caused an overestimation or underestimation of CA prevalence. Third, JECS did not collect data from alternative sources, such as child health systems or departments specializing in the diagnosis of CAs after 1 month of age. This is likely to have led to unidentified CA cases being ignored after 1 month of age. To identify overlooked CA cases, the JECS consortium plan to collect additional CA data on 2-year-old children receiving hospital treatment through the use of parent-report questionnaires. Finally, methodological differences between the JECS and other registries may have resulted in the differing estimates of CA prevalence.^39^ Once the JECS data become open access, the present authors recommend that two data reporting groups, for example, D-1M and D-OR, are used for the analysis of each CA. The number of omphalocele recorded at the 1 month data collection time-point may have been an overestimate, due to misclassification secondary to the similar Japanese terminology for omphalocele and umbilical hernia. Thus, for omphalocele, D-DELIVERY or D-AND data should be used to determine prevalence. There were some instances of misclassification in the JECS data. Since JECS is not a population-based registration program, the data cannot be used to generate definite CA prevalence rates. However, the demographic characteristics of the JECS pregnant women cohort are almost identical to those reported in a previous national survey.^7^

### Conclusions

The present report generated reliable data concerning the prevalence of major CAs in the JECS cohort.

## APPENDIX A. SUPPLEMENTARY DATA

eTable 1. Number of infants at each regional center according to maternal age at delivery

eTable 2. Number and proportion of congenital anomaly cases confirmed with ICD-10 code in the reporting pattern in the Kochi regional center

## APPENDIX B.

Members of the Japan Environment and Children’s Study (JECS), as of 2017 (principal investigator, Toshihiro Kawamoto): Hirohisa Saito (National Centre for Child Health and Development, Tokyo, Japan), Reiko Kishi (Hokkaido Regional Center for JECS, Hokkaido University, Sapporo, Japan), Nobuo Yaegashi (Miyagi Regional Center for JECS, Tohoku University, Sendai, Japan), Koichi Hashimoto (Fukushima Regional Center for JECS, Fukushima Medical University, Fukushima, Japan), Chisato Mori (Chiba Regional Center for JECS, Chiba University, Chiba, Japan), Shuichi Ito (Kanagawa Regional Center for JECS, Yokohama City University, Yokohama, Japan), Zentaro Yamagata (Koshin Regional Center for JECS, University of Yamanashi, Chuo, Japan), Hidekuni Inadera (Toyama Regional Center for JECS, University of Toyama, Toyama, Japan), Michihiro Kamijima (Aichi Regional Center for JECS, Nagoya City University, Nagoya, Japan), Takeo Nakayama (Kyoto Regional Center for JECS, Kyoto University, Kyoto, Japan), Hiroyasu Iso (Osaka Regional Center for JECS, Osaka University, Suita, Japan), Masayuki Shima (Hyogo Regional Center for JECS, Hyogo College of Medicine, Nishinomiya, Japan), Yasuaki Hirooka (Tottori Regional Center for JECS, Tottori University, Yonago, Japan), Narufumi Suganuma (Kochi Regional Center for JECS, Kochi University, Nankoku, Japan), Koichi Kusuhara (Fukuoka Regional Center for JECS, University of Occupational and Environmental Health, Kitakyushu, Japan), and Takahiko Katoh (South Kyushu/Okinawa Regional Center for JECS, Kumamoto University, Kumamoto, Japan).

## References

[1] Congenital anomalies fact sheet [homepage on the Internet]. Available from: http://www.who.int/mediacentre/factsheets/fs370/en/.

[2] Oliveira CI, Fett-Conte AC Birth defects: risk factors and consequences. J Pediatr Genet. 2013;2:85–90. 27625844 10.3233/PGE-13052 PMC5020963

[3] Gorini F, Chiappa E, Gargani L, Picano E Potential effects of environmental chemical contamination in congenital heart disease. Pediatr Cardiol. 2014;35:559–568. 10.1007/s00246-014-0870-1 24452958

[4] Vrijheid M, Martinez D, Manzanares S, Ambient air pollution and risk of congenital anomalies: a systematic review and meta-analysis. Environ Health Perspect. 2011;119:598–606. 10.1289/ehp.1002946 21131253 PMC3094408

[5] Virtanen HE, Adamsson A Cryptorchidism and endocrine disrupting chemicals. Mol Cell Endocrinol. 2012;355:208–220. 10.1016/j.mce.2011.11.015 22127307

[6] Hei MY, Yi ZW Environmental factors for the development of fetal urinary malformations. World J Pediatr. 2014;10:17–23. 10.1007/s12519-014-0449-1 24464659

[7] Michikawa T, Nitta H, Nakayama SF, ; Japan Environment and Children’s Study Group Baseline profile of participants in the Japan Environment and Children’s Study (JECS). J Epidemiol. 2018;28(2):99–104. 10.2188/jea.JE20170018 29093304 PMC5792233

[8] Kawamoto T, Nitta H, Murata K, ; Working Group of the Epidemiological Research for Children’s Environmental Health Rationale and study design of the Japan environment and children’s study (JECS). BMC Public Health. 2014;14:25. 10.1186/1471-2458-14-25 24410977 PMC3893509

[9] Copeland KT, Checkoway H, McMichael AJ, Holbrook RH Bias due to misclassification in the estimation of relative risk. Am J Epidemiol. 1977;105:488–495. 10.1093/oxfordjournals.aje.a112408 871121

[10] Duan R, Cao M, Wu Y, An empirical study for impacts of measurement errors on EHR based association studies. AMIA Annu Symp Proc. 2016;2016:1764–1773. 28269935 PMC5333313

[11] Japan Environment and Children’s Study (JECS) Study Protocol (ver. 1.4) [homepage on the Internet]; c2016. Available from: http://www.env.go.jp/chemi/ceh/en/about/advanced/material/jecs-study_protocol_14_en.pdf.

[12] Schechtman KB, Gray DL, Baty JD, Rothman SM Decision-making for termination of pregnancies with fetal anomalies: analysis of 53,000 pregnancies. Obstet Gynecol. 2002;99:216–222. 11814500 10.1016/s0029-7844(01)01673-8

[13] Suzumori N, Kumagai K, Goto S, Nakamura A, Sugiura-Ogasawara M Parental decisions following prenatal diagnosis of chromosomal abnormalities: implications for genetic counseling practice in Japan. J Genet Couns. 2015;24:117–121. 10.1007/s10897-014-9744-1 25082303

[14] International Clearinghouse for Birth Defects Surveillance and Research. Annual Report 2014 [homepage on the Internet]; c2014. Available from: http://www.icbdsr.org/wp-content/annual_report/Report2014.pdf.

[15] European surveillance of congenital anomalies. Prevalence Tables [homepage on the Internet]; c2016. Available from: http://www.eurocat-network.eu/AccessPrevalenceData/PrevalenceTables.

[16] Parker SE, Mai CT, Canfield MA, ; National Birth Defects Prevention Network Updated National Birth Prevalence estimates for selected birth defects in the United States, 2004–2006. Birth Defects Res A Clin Mol Teratol. 2010;88(12):1008–1016. 10.1002/bdra.20735 20878909

[17] AR C, S R. *Early management of myelomeningocele*. WB Saunders; 2001.

[18] Cockell A, Lees M Prenatal diagnosis and management of orofacial clefts. Prenat Diagn. 2000;20:149–151. 10.1002/(SICI)1097-0223(200002)20:2<149::AID-PD764>3.0.CO;2-U 10744509

[19] Hanaoka T, Tamura N, Ito K, ; other members of the Hokkaido Study on Environment and Children’s Health Prevalence and risk of birth defects observed in a prospective cohort study: the Hokkaido Study on Environment and Children’s Health. J Epidemiol. 2018;28(3):125–132. 10.2188/jea.JE20160108 29093352 PMC5821689

[20] Shiraishi M, Mishima K, Umeda H, Ueyama Y Clinico-statistical study of the cases of cleft lip and/or palate in the past 10 years at our department. Yamaguchi Med J. 2017;66:169 (in Japanese).

[21] Mai CT, Cassell CH, Meyer RE, ; National Birth Defects Prevention Network Birth defects data from population-based birth defects surveillance programs in the United States, 2007 to 2011: highlighting orofacial clefts. Birth Defects Res A Clin Mol Teratol. 2014;100(11):895–904. 10.1002/bdra.23329 25399767 PMC4631395

[22] Young DL, Schneider RA, Hu D, Helms JA Genetic and teratogenic approaches to craniofacial development. Crit Rev Oral Biol Med. 2000;11:304–317. 10.1177/10454411000110030201 11021632

[23] Lu XC, Yu W, Tao Y, Contribution of transforming growth factor alpha polymorphisms to nonsyndromic orofacial clefts: a HuGE review and meta-analysis. Am J Epidemiol. 2014;179:267–281. 10.1093/aje/kwt262 24243742

[24] Blanton SH, Cortez A, Stal S, Mulliken JB, Finnell RH, Hecht JT Variation in IRF6 contributes to nonsyndromic cleft lip and palate. Am J Med Genet A. 2005;137A(3):259–262. 10.1002/ajmg.a.30887 16096995

[25] Beaty TH, Murray JC, Marazita ML, A genome-wide association study of cleft lip with and without cleft palate identifies risk variants near MAFB and ABCA4. Nat Genet. 2010;42:525–529. 10.1038/ng.580 20436469 PMC2941216

[26] Hernández-Díaz S, Werler MM, Walker AM, Mitchell AA Folic acid antagonists during pregnancy and the risk of birth defects. N Engl J Med. 2000;343(22):1608–1614. 10.1056/NEJM200011303432204 11096168

[27] Butali A, Little J, Chevrier C, Folic acid supplementation use and the MTHFR C677T polymorphism in orofacial clefts etiology: an individual participant data pooled-analysis. Birth Defects Res A Clin Mol Teratol. 2013;97:509–514. 10.1002/bdra.23133 23670871 PMC3745533

[28] Kummet CM, Moreno LM, Wilcox AJ, Passive smoke exposure as a risk factor for oral clefts—a large international population-based study. Am J Epidemiol. 2016;183:834–841. 10.1093/aje/kwv279 27045073 PMC4851990

[29] Shaw GM, Lammer EJ Maternal periconceptional alcohol consumption and risk for orofacial clefts. J Pediatr. 1999;134:298–303. 10.1016/S0022-3476(99)70453-1 10064665

[30] Carmichael SL, Shaw GM, Ma C, Werler MM, Rasmussen SA, Lammer EJ; National Birth Defects Prevention Study Maternal corticosteroid use and orofacial clefts. Am J Obstet Gynecol. 2007;197(6):585.e1–585.e7; discussion 683–684, e1–e7. 10.1016/j.ajog.2007.05.046 18060943

[31] Jackson A, Bromley R, Morrow J, Irwin B, Clayton-Smith J In utero exposure to valproate increases the risk of isolated cleft palate. Arch Dis Child Fetal Neonatal Ed. 2016;101:F207–F211. 10.1136/archdischild-2015-308278 26408639

[32] Sukanya S, Bay BH, Tay SS, Dheen ST Frontiers in research on maternal diabetes-induced neural tube defects: past, present and future. World J Diabetes. 2012;3:196–200. 10.4239/wjd.v3.i12.196 23301121 PMC3538985

[33] Stothard KJ, Tennant PW, Bell R, Rankin J Maternal overweight and obesity and the risk of congenital anomalies: a systematic review and meta-analysis. JAMA. 2009;301:636–650. 10.1001/jama.2009.113 19211471

[34] Ornoy A Neuroteratogens in man: an overview with special emphasis on the teratogenicity of antiepileptic drugs in pregnancy. Reprod Toxicol. 2006;22:214–226. 10.1016/j.reprotox.2006.03.014 16621443

[35] Pettigrew SM, Bell EM, Van Zutphen AR, ; National Birth Defects Prevention Study Paternal and joint parental occupational pesticide exposure and spina bifida in the National Birth Defects Prevention Study, 1997 to 2002. Birth Defects Res A Clin Mol Teratol. 2016;106(11):963–971. 10.1002/bdra.23551 27891778 PMC6613649

[36] Marinković S, Bukarica S [Umbilical hernia in children]. Med Pregl. 2003;56(5–6):291–294. 10.2298/MPNS0306291M 14565056

[37] Bower C, Rudy E, Callaghan A, Quick J, Nassar N Age at diagnosis of birth defects. Birth Defects Res A Clin Mol Teratol. 2010;88:251–255. 20213697 10.1002/bdra.20658

[38] British Isles Network of Congenital Anomaly Registers. Congenital Anomaly Statistics 2012 England and Wales [homepage on the Internet]; c2014. Available from: http://www.binocar.org/content/Annual%20report%202012_FINAL_nologo.pdf.

[39] Dolk H Epidemiologic approaches to identifying environmental causes of birth defects. Am J Med Genet C Semin Med Genet. 2004;125C:4–11. 10.1002/ajmg.c.30000 14755428

