# Supplementary material for: Prevalence of Congenital Anomalies in the Japan Environment and Children’s Study
Source: J Epidemiol. 2019 Jul 5;29(7):247–56. doi: 10.2188/jea.JE20180014 (PMC6556438; doi:10.2188/jea.JE20180014)
Supplement: Supplementary file 1 [file je-29-247-s001.pdf]

eTable 1. Number of infants at each regional center according to maternal age at delivery

	Total	Hokkaido	Miyagi	Fukushima	Chiba	Kanagawa	Koshin	Toyama	Aichi	Kyoto	Osaka	Hyogo	Tottori	Kochi	Fukuoka	South kyushyu
Number	101,825	8,051	9,210	13,013	6,047	6,523	7,242	5,496	5,622	3,966	7,966	5,134	3,066	7,040	7,649	5,800
maternal age at delivery, years																
<20	895	38	84	127	60	68	76	19	40	30	120	38	19	59	62	55
20–24	9,304	653	1,268	1,467	527	449	566	334	381	193	942	424	250	549	616	685
25–29	27,964	2,145	2,859	3,924	1,706	1,574	1,902	1,458	1,454	924	2,155	1,327	824	1,981	1,969	1,762
30–34	35,936	2,984	3,055	4,460	2,105	2,344	2,590	1,986	2,110	1,504	2,586	1,845	1,118	2,531	2,804	1,914
35–39	22,978	1,848	1,619	2,565	1,406	1,669	1,719	1,421	1,350	1,070	1,794	1,237	714	1,600	1,818	1,148
≥40	4,736	381	322	468	243	417	389	277	287	245	369	262	141	320	379	236
unknown	12	2	3	2	0	2	0	1	0	0	0	1	0	0	1	0

eTable 2. Number and proportion of congenital anomaly cases confirmed with ICD-10 code in the reporting pattern in the Kochi regional center

		D-DELIVERY[a]		D-1M[b]		D-OR[c]		D-AND[d]	
Medical records	Confirmed	102	82.3%	113	83.7%	132	78.6%	83	91.2%
	Not confirmed	22	17.7%	22	16.3%	36	21.4%	8	8.8%
Total		124	100%	135	100%	168	100%	91	100%

ICD-10, International Statistical Classification of Diseases and Related Health Problems, 10th revision.

Confirmed and non-confirmed status expressed in terms of total number and percentage by categories.

[a] D-DELIVERY includes congenital anomalies reported only at delivery data collection.

[b] D-1M includes congenital anomalies reported only at 1 month data collection.

[c] D-OR includes congenital anomalies reported either at delivery or at 1 month data collection.

[d] D-AND includes congenital anomalies reported at both delivery and at 1 month data collection, only at delivery, and only at 1 month data collection.
